# Dental Periodicals

**Published:** 1856-12

**Authors:** Jas. Taylor


### DENTAL PERIODICALS.

The periodical literature of our profession has I think a more direct connection with its advancement than a large majority are willing to concede. This may be and I think is from a want of proper information and reflection on the subject.

It is now an established fact that to keep pace with any department of science its periodical literature must be constantly consulted, every new improvement or valuable suggestion first meets the public eye through this channel, and to wait for regular and systematic publications embodying all recent discoveries would be like waiting for the stage coach to carry us to the next town; when the railroad cars would take us there any hour we might wish to go.

I might say that new books are now only necessary for the compiling and arranging of recent and valuable facts, which have been loosely scattered by the periodical press. Like the collections of a museum they need arranging; and like in the latter case many specimens may need much polishing or trimming before they are ready to take their place on the shelf along with the class to which they belong.

There are many in our profession who I fear prefer waiting for this trimming, polishing and shelving before they think it worth while to make an examination. When the museum is prepared and the music has struck up, they step in, gloved and equiped for a general survey, with opera-glass in hand they glance over the binding and if it suit they exclaim "how beautiful!" My propositions are first, that our dental periodicals are essentially necessary to the advancement and elevation of our profession; and second, that every member of the profession is under obligation to sustain them.

These propositions appear so self-evident, that argument to prove them is useless, and but for some experience which has met me so often with its stern reality, I should only state these facts, believing in their full reception.

If dental science is progressing, and no sane man can doubt this, something must cause this progress. It can hardly be an isolation of its members each imparting their experience to the other as chance may throw them together. Many centuries of experience have proved that but slow progress is made in this way, many of us who have been in practice twenty to thirty years know how little and restricted was this professional intercourse before the introduction of our periodicals. The fact is, up to within this period but few were disposed to let others profit by their experience.

Our dental periodicals have shaken out the old secrets of the profession and scattered them broadcast over the land; and the most confirmed *Fogy* whether he reads or not is really benefitted by it. It must be almost impossible to practice the profession now and not feel the influence and be incited to further excellence by the improvements which are written of, spoken of, and almost daily seen.

I do not wish to detract one iota from the venerated pioneers of our Profession in this country and Europe. To their skill may we attribute that impetus which was first given to dental science. Before their skill prejudice gave way, and thus was prepared a public sentiment which demands a dental literature which shall be on a par with that of general medicine. They were however differently situated from what we are now, and to say because they did their duty nobly to the profession, we could be doing the same to follow in all respects their footsteps would be requiring nothing for increased light and advantages. If fifty in the profession were not able to sustain one periodical, five thousand should not make that excuse.

Every man is somewhat indebted to his profession and he has but a poor appreciation of his duties, if he supposes *it* must make him, while he will not take any interest in *its* elevation.

I have thought that no set of publishers have more serious obstacles to meet than those who publish and edit for us our dental periodicals. In the first place the number of the profession is not sufficiently large to give a very extensive circulation, and nearly one half who receive such works are constantly shifting about; so that at the end of the year one half of those he may consider subscribers, may have in their estimation thrown off all obligation by shifting their location, leaving the remaining numbers of the work as refuse paper for the postmasters' use. To those who are by some personal sacrifice aiming to lay deep and broad the foundations of our science without any consideration in the way of business, this is a sore evil. Those who issue periodicals for the advertising medium it affords them, cannot feel this to the same extent. If their advertisement is seen by any one in the profession at all the different points to which it is sent, the great object is attained. They have made known their business and where they may be found, and subscription to their work is only the secondary consideration.

There is a difficulty here to be met in the publication of regular dental periodicals which I fear has not been duly considered. The question might be stated as follows, are we to look for our dental periodicals to those engaged in the manufacture and sale of dental materials; or to the profession itself? The former may and *can* give us cheaper publications, but would the profession do well and act wisely to rely upon them. Suppose the druggists of the United States were to control the medical publications? Let every manufacturer of drugs issue a medical journal and charge for the same about half what the regular practitioner could afford the same for and what would be the result!

I am now just throwing out thoughts for the consideration of the profession, as well as those supplying the profession with its literature, and would not be considered as reflecting on those engaged in publishing periodicals as advertising mediums.

Time was when the profession's interest may have been promoted by such a course. There are however epochs in a profession's advancement as well as a nation's, and it becomes us to take advantage of such eras and select that vessel in which to sail, which will most surely lead to a successful and prosperous destination. Having had some experience on what I now write of, I hope my remarks will be received as they are given with the best motives, having only in view the good of the profession.

An editor's correspondence unfolds many curious features in science as well as politics, a few rare specimens only have I kept for future reference, one which I was tempted to publish at the time it was received* will answer to illustrate the difficulties which dental journalists have to encounter. The letter is as follows: I omit names, locality, date, &c. The sentiment is all that is necessary.

"*Dear Sir:* Yours of the 10th is before me and I have overhauled a number of *stacks of periodicals,* pamphlets, &c., and can only find 5 nos. of your journal, viz. vol. 7 nos. 1, 2, 4, vol. 6 no. 4, vol. 5 no. 2, so you see they are rather irregular. I will send them to you with pleasure if you say so, but I have not the numbers you speak of, if I have, it is not known to me, I do not remember anything about them, therefore

*When I assumed the editorial of the Register I determined to exclude all letters of commendation as well as those of censure. It is some pleasure, now to know that the latter were much less frequent than the former, that a large majority of the Profession live on the sweets of life and extend a happy smile to the Profession at large, while only a few suffer the acids of their Laboratory to get on their own teeth and as the result of their negligence grow cross and bite and snarl at every thing in their way. J. T.

conclude I did not get them. I am *constantly receiving Journals of similar character from different parts of the country and have written for some of them, but I seldom read an article, merely glance at the index* and *pass* them over to the file for future reference if need be.

I have *never been a subscriber to any dental publication, yet they come free with the exception of postage. Twenty-eight years of experience in the profession, has taught me many things which I cannot expect to get from journals.*

The lamented Dr. —— of —— was my particular friend and correspondent. We have been so since 1828, I have never found one in the profession who could originate such valuable improvements in our profession as that valuable Brother. Yet his enthusiasm would sometimes lead him into error, he did not wish to be baffled even by impossibilities, he adopted plans of cure which proved to be more useful than healthy, his benevolent heart led him to try a curative process which his head could not (in the end) recognize as healthy, examples could be given to prove this, if time would permit. Repectfully yours."

The italics are mine. The letter, however, speaks for itself, he is so " constantly receiving journals, *free* it that would be a great tax to pay for one, he with many others will never pay for knowledge when it comes free. But why is it that this " experience" gained in *twenty-eight years practice* is not made subservient to the profession's advancement ?

The Register had been regularly mailed to this professional brother for three years with a request if he did not want it to say so by returning it. In the lapse of time only two numbers of the first two volumes could be found. Three numbers of the last volume were " still on file." I am kindly informed, if I want them, they will be sent to me. I could not help but think that this brother's " stacks of periodicals" cost somebody some money, and that if somebody else had paid for them, they would have been more carefully kept ; yet a few dollars out of a practice worth fifty dollars a day could not well be spared and I let the *case* slide.

How often do I hear this and that one say, "I take so and so and they cost me nothing?" Now to you who are able, I say, Out, on such meanness, take one or two Journals and pay for them, I have been now over "twenty-eight years in practice;" and I find that there is so much in the "Journals" which my "experience" has not taught *me*, that I cannot afford to do without them. I hope I shall never lose the relish for that delightful, sweet and yet silent interchange of thought which the press affords, and which is doing so much for every department of science. JAS. TAYLOR.

